# Next generation sequencing reads comparison with an alignment-free distance

**DOI:** 10.1186/1756-0500-7-869

**Published:** 2014-12-03

**Authors:** Emanuel Weitschek, Daniele Santoni, Giulia Fiscon, Maria Cristina De Cola, Paola Bertolazzi, Giovanni Felici

**Affiliations:** Department of Engineering, Roma Tre University, Via della Vasca Navale 79, 00146 Rome, Italy; Institute of Systems Analysis and Computer Science “A. Ruberti”, National Research Council, Via dei Taurini 19, 00185 Rome, Italy; Department of Computer, Control, and Management Engineering “Antonio Ruberti”, Viale Ariosto 25, 00185 Rome, Italy; IRCCS Centro Neurolesi “Bonino-Pulejo”, S.S.113 Via Palermo C/da Casazza, 98123 Messina, Italy

**Keywords:** Sequence analysis, Next generation sequencing, Alignment-free

## Abstract

**Background:**

Next Generation Sequencing (NGS) machines extract from a biological sample a large number of short DNA fragments (*reads*). These reads are then used for several applications, e.g., sequence reconstruction, DNA assembly, gene expression profiling, mutation analysis.

**Methods:**

We propose a method to evaluate the similarity between reads. This method does not rely on the alignment of the reads and it is based on the distance between the frequencies of their substrings of fixed dimensions (*k*-mers). We compare this alignment-free distance with the similarity measures derived from two alignment methods: Needleman-Wunsch and Blast. The comparison is based on a simple assumption: the most correct distance is obtained by knowing in advance the reference sequence. Therefore, we first align the reads on the original DNA sequence, compute the overlap between the aligned reads, and use this overlap as an ideal distance. We then verify how the alignment-free and the alignment-based distances reproduce this ideal distance. The ability of correctly reproducing the ideal distance is evaluated over samples of read pairs from *Saccharomyces cerevisiae*, *Escherichia coli*, and *Homo sapiens*. The comparison is based on the correctness of threshold predictors cross-validated over different samples.

**Results:**

We exhibit experimental evidence that the proposed alignment-free distance is a potentially useful read-to-read distance measure and performs better than the more time consuming distances based on alignment.

**Conclusions:**

Alignment-free distances may be used effectively for reads comparison, and may provide a significant speed-up in several processes based on NGS sequencing (e.g., DNA assembly, reads classification).

**Electronic supplementary material:**

The online version of this article (doi:10.1186/1756-0500-7-869) contains supplementary material, which is available to authorized users.

## Background

The development of Next Generation Sequencing (NGS) machines allows the extraction of an extremely large amount of reads (i.e., short fragments of an organism’s genome) at low cost. The length of such reads is very small when compared to the length of a genome: it may range from 40 to 300 base pairs (bp) (i.e., characters), while the length of a simple genome (e.g., bacteria) is in the order of millions base pairs (Mbp). Three main NGS technologies are currently used [1]: Roche 454, Illumina, and Ion Torrent. At present, Illumina technology performances are 40 gigabase pairs (Gbp) per day at a low cost per bp [illumina.com] with reads average length of 70 bp; Roche 454 performances are 1 Gbp per day at a higher cost with reads average length of 250 bp [454.com]; Ion Torrent machines produce reads of 200 bp with a throughput of 5 Gbp per day at a low cost per bp [2].

DNA assembly can be defined as the reconstruction of a genome, starting from a large number of short overlapped fragments (*reads*) obtained by a *sequencing* operation. The length of each read and the number of reads are determined by the type of sequencer. The complexity of the assembly process stems from the length and number of the reads: longer reads are easier to be assembled, while a larger number of short reads requires a higher computational effort, although providing more information. Typically, the number of reads produced by NGS experiments reaches several millions or more, depending on the sequencing coverage and on its depth. The use of NGS machines results in much larger sets of reads to be assembled, posing new problems for computer scientists and bioinformaticians, whose task is to design algorithms that align and merge the reads for an effective reconstruction of the genome (or large portions of it) with sufficient precision and speed [3].

Many competing algorithms have been developed for DNA assembly: a comprehensive comparison of recent and well-established ones can be found in [4] and [5], where these methods are tested on common benchmarks. The assembly problem is proven to be NP-hard [6] and several heuristic algorithms have been proposed. Algorithms for DNA assembly are based on two main approaches: overlap graphs (e.g., [7]) and De Bruijn Graphs [4]. In an overlap graph each read corresponds to a node, and the overlaps between read pairs - that define the weights of the arcs - are usually computed by means of alignment methods; an assembly is derived from an Hamiltonian path in this graph. In the De Bruijn Graphs approach, reads are represented on a graph whose nodes and edges are nucleotide subsequences of length *k* (called *k*-mers) [8]; an edge corresponds to an overlap between two nodes. The assembly is found searching for an Eulerian cycle in this graph and it is represented by a sequence of edges. Several assembly software tools (e.g., ABySS [9], Velvet [10], and SoapDeNovo [11]) use subsequences of fixed dimensions (*k*-mers) for building the De Bruijn graph. These and other well-established assembly algorithms (e.g., Ssake, Sharcgs, Vcake, Newbler, Celera Assembler, Euler, and AllPaths) are described and compared in [12]. We note that the role of *k*-mers in the assembly approaches based on De Bruijn graph is substantially different from the role they play in the the definition of the alignment-free distance described later. In fact, the De Bruijn graph uses *k*-mers as nodes of the graph and does not consider their frequency, while our approach is based on the frequency of *k*-mers to assess reads similarity.

A large number of these algorithms - in particular, those using the overlap graphs - are based on the similarity between reads. Such a similarity is the main way to assess whether two reads may be overlapped in the reconstruction process or not. In these approaches, such a measure is hence required to compare each read pair, generating a number of comparisons that is potentially quadratic in the number of reads. Therefore, it is extremely important to develop methods that can quickly establish whether two reads are similar or not.

In this paper, we focus on alignment-free techniques that have been proven to be effective in sequence analysis [13, 14]. These techniques can be classified into two main groups: methods based on sequence compression and methods that rely on subsequence (oligomers) frequencies [13]. The aim of the methods belonging to the first group is to find the shortest possible description of the sequence. They compute the similarity of the sequences by analyzing their compressed representations. Currently available methods are based on the Kolmogorov complexity [15] and on Universal Sequence Maps [16]. An extensive review can be found in [17]. The methods based on oligomers frequencies rely on the computation of the substring frequencies of a given length *k* in the original sequences, called *k*-mers. Here, the similarity of two sequences is based only on the dictionary of subsequences that appear in the strings, irrespective of their relative position [17].

The alignment-free distance adopted in this study is inspired to the *k*-mer frequency analysis [18], where the frequencies of the *k*-mers are represented in a real vector, and hence they are easily tractable in a mathematical space: the distance between two reads is obtained by the distance between their frequency vector representations. A simple and easy way to compute a distance measure is the Euclidean distance, although others may be used (e.g., the *d*2 distance of [19]). The goal of this paper is to evaluate the reliability of an alignment-free distance for read pairs similarity and to compare it with respect to other read-to-read distances that are based on global or local alignment of the two reads.

The paper is organized as follows.

In section *Methods*, we provide sufficient background for the main methods and techniques used in the paper: the different adopted read pairs distances are described (subsections *Bowtie distance*, *Needleman-Wunsch edit distance*, *The Blast alignment distance* and *Alignment-free distance on tetramer frequencies*). Following, we outline the rationale of threshold predictors and the way they are computed from data. Section *Results and discussion* describes the experimental design and its results. First, we delineate the data sets extraction and the experimental procedure (subsection *Data sets and experimental settings*). Then, we consider the computational performances of the different distances (subsection *Computational time analysis of the threshold predictors*), how they correlate among each other (subsection *Pearson correlation among distances*), their prediction performances over the training sets with the support of ROC curves and AUC indicators (subsection *Performance analysis of the threshold predictors*), and their predictive results for a cross validation evaluation scheme (subsection *Cross validation performances of the*AF*threshold predictor*). Finally, we provide discussion of the results (subsection *Final discussion*). In section *Conclusions* we delineate the conclusions and the perspectives of the work.

## Methods

We consider a straightforward implementation of the alignment-free distance, based on the euclidean distance of the frequency distribution of *k*-mers (i.e., substrings composed of *k* consecutive bases) in the two reads. Such a distance, referred to as AF in the following, is very simple to compute and requires linear time in the dimension of the reads. As far as the choice of the length of the oligomers, we adopt *k*=4 (tetramers) as in many references this value has been confirmed to provide an ideal balance between the length of the oligomers and their number, when the sequences are expressed in the (A, C, G, T) alphabet [20–22]. AF is compared with respect to two methods to measure DNA string similarity that are based on sequence alignment: the Needleman-Wunsch edit distance (NW) and the Blast alignment algorithm (BL).

Both methods require quadratic time in the length of the reads. Their choice is motivated by the fact that the first is a global alignment method, i.e., it searches for the best alignment of the complete reads, while the second is a local alignment, i.e., it searches for the longest possible portion that is aligned well within the two reads. Therefore, their choice covers the two main approaches used in computing alignment-based distances.

To perform a proper comparison among AF, NW, and BL we adopt the following test. First, we assume the existence of an *ideal distance*, i.e., the distance that is given by the degree of overlapping of reads that have been aligned on their known reference genome. Second, we verify the ability of the three distances in approximating this ideal (target) distance. Given a pair of reads, such an approximation is measured by the ability of predicting the value of the target distance using the value of the predicting one. This assumption is based on the fact that an assembly method that uses the target distance to evaluate the opportunity of overlapping two reads would result in a extremely satisfactory assembly.

To align the reads over the original sequence, we use the well-established Bowtie algorithm [23, 24]; two reads receive a maximum distance value if they do not overlap over the reference sequence; otherwise, they receive a distance inversely proportional to their degree of overlapping over the sequence (e.g., they would have minimum distance if they are aligned in the same position by the Bowtie algorithm). Given two reads, we define such a value their *Bowtie distance* (BT in the following). We refer to BT as the *target* distance and either to AF, or NW or BL as the *predictor* distance. A *threshold predictor* is a mapping between the values of the target distance and the values of the predictor distance; in other words, it assigns to each value of the target distance, say *α*, a value of the predictor distance, say *β*. Informally, we may define the threshold predictor as a mapping *m* such that *m*(*α*) = *β*. Then, the target distance between two given reads is predicted to be below *α* when the predictor distance between the same two reads is below *β*.

According to the above definition, for each value of the target distance the threshold predictor may incur in errors in terms of false positive and false negative predictions. The quality of the threshold predictor is given by the error distribution over the predicted values of the target distance.

To test our method, we consider DNA sequences of three different organisms: *Saccharomyces cerevisiae*, *Escherichia coli*, and *Homo sapiens*. Publicly available sets of NGS reads for the three reference sequences are used. Each experiment is based on a large sample of read pairs, from which the best possible threshold predictor (among AF, NW, or BL) of the BT distance value is computed. The precision of the predictors is evaluated building ROC curves both on the samples of read pairs used to identify the best predictors (training data), and on other samples from the same set of read pairs not used for training (testing data). The results show how AF performs very well as a threshold predictor for BT; its performances are indeed better than those of NW and comparable to those exhibited by BL. Furthermore, both NW and BL are much more demanding in terms of computing time when compared with respect to AF.

### Bowtie distance

The Bowtie distance (BT) is obtained after computing the alignments of the reads to the reference genome with the Bowtie algorithm [23, 24]. Bowtie is able to align reads to the reference genome at a very high speed (25 million reads of 35 bp length per hour).

Prior to the computation of the alignments, Bowtie builds an index of the reference genome with a Burrows-Wheeler approach. Two versions of Bowtie are available: Bowtie 1 [23] and Bowtie 2 [24]. The first one is optimized for short genomes, the latter for longer ones and supports gapped, local, and paired-end alignment modes. For each read, the alignment position in the reference genome is obtained after running the Bowtie algorithm. Given two reads *r*_1_, *r*_2_ we define the Bowtie distance as follows:


where ∇(*r*_1_,*r*_2_) is the number of overlapped positions of *r*_1_ and *r*_2_, *λ*_1_ is the length of *r*_1_, and *λ*_2_ is the length of *r*_2_. If multiple alignments of the same reads are present, their average is used.

### Needleman-Wunsch edit distance

The Needleman and Wunsch algorithm (NW) [25], based on dynamic programming, is commonly used to perform a global alignment of two sequences. The algorithm time complexity is quadratic with respect to the lengths of the two sequences (*N* and *M*) to be aligned (*O*(*n* ∗ *m*), where *n* and *m* are the number of bases in the two reads). The NeoBio [26] Java implementation of the NW algorithm is adopted for performing the distance evaluation experiments. We adopt following parameter settings for the NW algorithm:

-  +1 for the reward of a match (i.e., a substitution of equal characters);
 -1 for the penalty of a mismatch (i.e., a substitution of different characters);
 -1 for the cost of a gap (i.e., an insertion or deletion of a character).

We use the above-mentioned configuration in order to assign an equally balanced score for a match (+1), a mismatch (-1), and a gap (-1). For further details we point the reader to the NeoBio documentation [26]. The NW distance is obtained from the Needleman-Wunsch score in two steps. First, the score is subtracted to its maximum possible value (perfect alignment) in order to obtain null distance in case of equal sequences and large distance for different ones; then, it is normalized between 0 and 1 to ease the comparisons with the other measures.

### The Blast alignment distance

The Basic Local Alignment Search Tool (Blast) [27] is used to compare a query sequence with respect to a library or database of sequences. Blast adopts an heuristic approach that is less accurate than other methods, but much faster. The Blast time complexity is also quadratic (*O*(*n* ∗ *m*) where *n* and *m* are the lengths of the two reads to be aligned). It is worth noting that this is the same time complexity as other algorithms, including the NW global alignment. However, given the heuristic nature of the algorithm, the statistically significant elimination of High-scoring Segment Pairs (HSPs) and words is used. In this way, Blast significantly reduces the amount of computation, running much faster than its worst case time complexity. In this work, we use Blast2 that is the Blast version to simply align two sequences. The Blast implementation available in [28] was adopted for computing the Blast scores and the Blast expected values between the considered read pairs. The parameters adopted for the runs are described in Table 1: we turn off the masking parameter, which filters out low complexity and high frequency regions (e.g., repetitive parts) of the genomic sequence. The final Blast distance (BL) is obtained by subtracting the Blast score to its maximum value and normalizing it between 0 and 1 (given the fixed and equal size of the sequences, the Blast expected values resulted to be perfectly log-correlated with the Blast scores).

**Table 1:** **Blast parameters setting**

Parameter	Value	Description
-p	blastn	Blast program for nucleotide sequences
-F	F	Masking and filtering off
-w	4	Windows size
-i	r1.fas	First input filename
-j	r2.fas	Second input filename
-m	8	Alignment view set to tabular output

### Alignment-free distance on tetramer frequencies

We provide a simple sketch of the alignment-free distance computation used in this paper, mainly based on [13]. The frequencies of each substring of length 4 (also called *tetramers*) are computed by counting the occurrences of the substrings in the read with a sliding window of length 4, starting at position 1 and ending at position *n* - 4 + 1, where *n* is the length of the read. For the alphabet composed of the four symbols (A,C,G,T) we have a total of 4^4^ = 256 different tetramers and thus each read is represented by a vector of 256 real numbers between 0 and 1. The choice of tetramers is motivated by [20–22], which confirm the ideal balance between the length of the oligomers and their number. Given two reads, the Euclidean distance between their associated frequency vectors is an inverse measure of the similarity of the two reads, and we refer to it as the AF distance between the two reads. An efficient Java implementation of the alignment-free frequency vector computation and representation was developed for computing the AF distance between the available read pairs. The related algorithm is linear with respect to the length of the reads and is available at dmb.iasi.cnr.it/ngs_distances.php.

### Threshold distance predictors

Our goal is to show how the AF distance between two DNA reads can approximate the BT distance, taking into account a tolerable degree of accuracy. In greater details, we aim to show that AF approximates BT as well as NW or BL distances, although less computationally demanding.

For a formal definition of *threshold predictor*, we need some additional notation. Let *r*_1_ and *r*_2_ be two generic reads coming from a DNA sequencing operation, and *d*_1_(*r*_1_,*r*_2_), *d*_2_(*r*_1_,*r*_2_) be two alternative read-to-read distance functions. Given a vector *α* of dimension *m*, *α* = (*α*_1_,*α*_2_,…,*α*_*m*_), a *threshold predictor* of *d*_1_(·,·) by *d*_2_(·,·) is determined by a vector *β* = (*β*_1_,*β*_2_,…,*β*_*m*_). Given two reads *r*_1_,*r*_2_, the prediction on *d*_1_(·,·) is obtained by the following rule:


The threshold predictor depends on the choice of the value of *d*_1_(·,·) in the vector *α*. We are indeed interested in the prediction only if *d*_1_(*r*_1_,*r*_2_) is below a certain value based on the value of *d*_2_(*r*_1_,*r*_2_), and would like this prediction to be precise for small reference values (i.e., those contained in the vector *α*).

As anticipated, we have BT as *target* distance (i.e., *d*_1_(·,·)) and the other distances as *predictors* (i.e., *d*_2_(·,·)). Since the original sequence is unknown, it is not always possible to compute the BT distance. Hence, we focused on predicting the BT distance by means of NW, BL or AF. In this context, a threshold predictor which is precise for small values in *α* turns out to be very useful. Thereby, we are not interested in predicting whether two reads are far from each other: we only want to know if they are close to each other or not.

A proper way to evaluate the quality of a threshold predictor is to measure its errors over one or more samples of read pairs where all distances are known. For each value *α*_*i*_ we measure the *true positive* (i.e., read pairs that are below *α*_*i*_ according to the target distance and are predicted to be below *α*_*i*_ by the threshold predictor) and *true negative* (i.e., read pairs that are above *α*_*i*_ according to the target distance and are predicted to be above *α*_*i*_ by the threshold predictor) rates associated with the above rule, and from this derive standard performance indicators such as ROC curves and AUC values [29].

Read pair samples are used also to identify and test good threshold predictors. Given the value *α* = (*α*_1_,*α*_2_,…,*α*_*m*_), we compute for a sufficiently large set of candidate values *β* = (*β*_1_,*β*_2_,…) the true positive and the true negative rates, construct the associated ROC curve for each value *α*_*i*_, and derive the corresponding AUC value. If the AUC value is good enough, we identify the value *β*_*j*_ that provides the largest combination of true positive and true negative rates, and adopt that for *α*_*i*_. Then, a complete threshold predictor is obtained by repeating this operation for each *α*_*i*_,*i* = 1,*m*.

The measure of a distinct precision value for each level of the target function enables to evaluate the reliability of the predictors there where it is needed. Clearly, the validity of a *threshold predictor* depends on the quality and the representativeness of the samples used to train (e.g., to derive the predicting vector *β*) and test the method. For the latter we adopt a standard cross-validation approach: first, the read pairs are sampled in disjoint sets; then some of these sets are used for training (e.g., derive the best values of *β* for the given values of *α*) and others are used for testing (measure the error of the so obtained threshold predictor). In several iterations, the role of training and testing samples is exchanged in order to mitigate the potential bias associated with the sampling.

The set of reference values *α* (for the target distance) and *β* (for the predictors) that have been used for the experiments are the values that separate the *percentiles* of the read pair distance distribution. In this way, we have that the first component of the *α* vector is larger than 1*%* of the sampled read pairs, the second is larger than 2*%*, and so on (similarly for the *β* vector). This allows to sample the whole variation range of the normalized distances, obtaining a finer granularity in the portions where the density is higher. According to this choice, both *α* and *β* are vectors composed of 100 real values between 0 and 1 in non-decreasing order. Clearly, this choice may be changed with equally spaced intervals without a significant effect on the results, once the proper granularity of the intervals has been identified.

### Data sets and experimental settings

In this subsection, we describe the adopted NGS data sets and the experimental setting. Three different organisms are taken into account, *Saccharomyces cerevisiae* (commonly known as yeast), *Escherichia coli*, and *Homo sapiens* (commonly known as human).

We design and apply the following experimental procedure:

-  *N* reads are downloaded from the NCBI Sequence Read Archive (SRA) database [30], or Chang Gung University, Department of Parasitology, College of Medicine (CGU) [31], and different NGS platforms;
-  By using the Bowtie algorithm [23, 24] the reads are aligned to the corresponding reference genome ( [32, 33]);
-  From the resulting alignments a random selection of *rs* reads is computed and a reverse complemented representation is calculated, obtaining a total of *rtot* reads;
-  Out of all the possible pairs of different reads from this set, six subsets are selected, each with *rp* read pairs. In order to have half of the set with non overlapping reads (e.g., maximum Bowtie distance) and the other half with a variable degree of overlap, the random selection of these six subsets is controlled;
-  The four distances are then computed for each pair in the set: the Bowtie Distance BT, the Needleman and Wunsch edit distance NW, the Blast score BL, and the alignment-free distance AF over the tetramers (i.e., substrings of length 4);
-  These measures are all turned into proper distances (see section Methods) ranging from 0 to 1 with 0 corresponding to equal sreads and 1 corresponding to maximally different reads.

In the following the six datasets are referred as YA, YB, …, YF (Y as in yeast), EA, EB, …, EF (E as in E. coli), HA, HB, …, HF (H as in human).

A compact summarized overview and description of the data sets is given in Table 2.

**Table 2:** **Compact overview of the datasets**

	Datasets		
	Yeast	E. coli	Human
**Genome length**	12.1 Mb	4.6 Mb	3.2 Gb
**Sequencing machine**	Illumina HiSeq	Roche 454	Illumina GA II
**Database**	NCBI SRA	CGU	NCBI SRA
**Accession number**	ERX191563	-	SRX013970
**Run id**	ERR216898	-	SRR031057
**Number of downloaded reads (** ***N*** **)**	3,551,079	436,142	14,267,012
**Avg. reads length** ***±*** **st.dev**	100 ±6	235 ±4	75 ±5
**Total base pairs**	355.0 M	102.5 M	1.1 G
**Random selection of aligned reads (** ***rs*** **)**	54,860	100,000	183,672
**Total number of selected reads (** ***rtot*** **)**	109,720	200,000	367,344
**Read pairs in each subset** ***rp***	1 M	200,000	1 M
**Source chromosome**	chr1	-	chr1

It is worth noting that the choice from different platforms (Roche 454, Illumina GA II, and Illumina HiSeq) stems from three main reasons: first, we want to test our methods on different read lengths; second we aim to show that the performances of our distance are independent from the selected sequencing technology; lastly, these platforms are the most common ones.

## Results and discussion

The main goal of this work is to provide evidence that the *AF* distance is a suitable approach to approximate BT distance. We apply AF to the three different data sets described in subsection Data sets and experimental settings, showing the performances with respect to other two alignment based algorithms (NW, BL). As a first step, we compute the Pearson correlation coefficients among the distances in the read pairs samples; then, we compute the ability of each measure to predict BT distance at given BT thresholds, by ROC curves and the corresponding AUC values. We verify the consistency of the predictions by a cross validation scheme detailed in the following sections.

### Computational time analysis of the threshold predictors

All software implementations of BT, NW, BL, and AF are run under a 64 bit linux environment (kernel 2.6.26-2-amd64) with a 64 bit Oracle Java Virtual Machine (version 1.7.0_09) on an Intel Core i7 920 2.67 GHz processor with 24 GB RAM memory, 1 TB sata 7200rpm hard disk, and a Debian GNU Linux 5.0.10 operating system.

In Table 3 we show the computational time of the different threshold predictors on 1.00 E+4, 1.00 E+6, and 1.00 E+8 read pairs. The time was recorded by means of the *time* linux utility, which provides user (i.e., the actual cpu time spent for computation), system (i.e., time spent for system calls, e.g., input/output), and elapsed (i.e., real time between invocation and termination) times.

**Table 3:** **Computational time analysis**

Reads pairs	1.00 E+4			1.00 E+6			1.00 E+8		
Time [sec]	User	System	Elapsed	User	System	Elapsed	User	System	Elapsed
AF	2.09	0.44	1.95	91.42	36.89	126.96	10230.33	3530.08	13541.00
NW	11.98	0.42	11.74	1466.49	34.03	1501.03	362794.34	3686.95	365785.00
Blast	122.21	81.14	199.80	10203.07	7434.90	18747.00	1418254.45	946049.60	2365124.00

The results highlight the much lower running time requirements of the AF distance. From our experimental results we see that the running time of AF is approximately 10 times smaller than NW, that is in turn 10 times smaller than BL.

Regarding memory requirements and consumption, we note that if the algorithms compute the distance measures by loading all *n* ∗ *n* read pairs of average length *l* into memory, then they will require *l* ∗ *n* bytes (online implementation); else if they perform the computation separately for each read pair, then the memory consumption is 2*l* bytes (offline implementation).

### Pearson correlation among distances

An initial comparison among the four distances is based on the analysis of the correlation coefficients of one distance with the others, over a sufficiently large sample of read pairs. The matrices in Tables 4, 5 and 6 report the Pearson correlation values between the four read-to-read distances for the three organisms.

**Table 4:** **Pearson correlation matrix between the four read-to-read distances for Yeast - YA**

	BT	NW	BL	AF
**BT**	1.00	0.45	0.81	0.63
**NW**	0.45	1.00	0.48	0.52
**BL**	0.81	0.48	1.00	0.61
**AF**	0.63	0.52	0.61	1.00

**Table 5:** **Pearson correlation matrix between the four read-to-read distances for Human - HA**

	BT	NW	BL	AF
**BT**	1.00	0.68	0.72	0.67
**NW**	0.68	1.00	0.73	0.72
**BL**	0.72	0.73	1.00	0.63
**AF**	0.67	0.72	0.63	1.00

**Table 6:** **Pearson correlation matrix between the four read-to-read distances for E. coli -EA**

	BT	NW	BL	AF
**BT**	1.00	0.76	1.00	0.95
**NW**	0.76	1.00	0.76	0.82
**BL**	1.00	0.76	1.00	0.95
**AF**	0.95	0.82	0.95	1.00

For each organism, the correlations are computed in one of the six available samples. Similar results are obtained on the other samples (not shown). It is of interest to analyze the correlation of the predictor distances (NW, BL, AF) with the target distance BT. First, we note that the correlations measures are significantly different in the three organisms; in yeast the NW distance is extremely poorly correlated with BT, while such a correlation improves for E. coli and human; the correlation between AF and BT is also weaker in yeast than in the other two organisms. The BL correlation with BT appears to be the higher among the three predictors. Moreover, it is evident that BT distance is almost perfectly reproduced from the predictors BL and AF in E. coli, then followed by yeast and human.

We could not found our conclusions only on the correlations values. Indeed, the requirement of a linear dependence between target and prediction distances may be a biased condition for the existence of the BT threshold predictor. Correlation represents an average similarity over the whole scale of the target, whereas some (small) reference values of the target distance need to be predicted with higher accuracy. Hence, more appropriate evaluations reported on the following sections are used.

### Performance analysis of the threshold predictors

In this section, we analyze the performances of the three predictors on the target distance. As above-mentioned, we consider 100 intervals of the target BT distance corresponding to the percentiles of its distribution, and identify by exhaustive inspection the percentiles of the predictor distance that minimize the prediction error. Such an analysis is performed by means of ROC curves, where we plot the true positive rate against the false positive rate for a given percentile of the target distance, with the percentiles of the predictor distance varying from 1 to 100. We recall that an ideal ROC curve contains the point (0,1) and therefore the area under the ROC curve (AUC) has value 1. Smaller values of AUC represent poorer prediction performances, and, in general, an AUC value is usually considered very good when in the proximity of 0.9.

**Figure 1:**
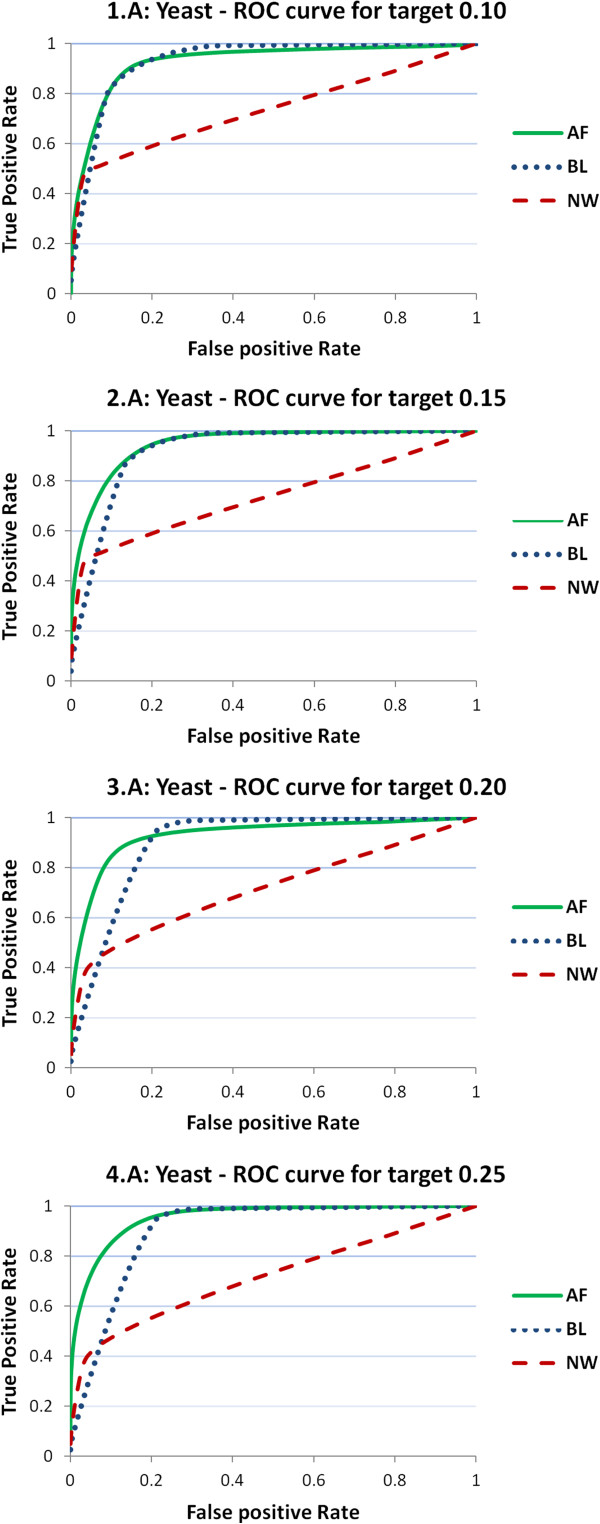
**ROC curves obtained for threshold predictors of yeast samples.** ROC curves obtained from threshold predictors of four different reference values of the target BT distance (0.10, 0.15, 0.20, 0.25). The charts report the ROC curves for the three predictor distances (NW, BL, AF). Results are provided on samples for yeast (YA).

**Figure 2:**
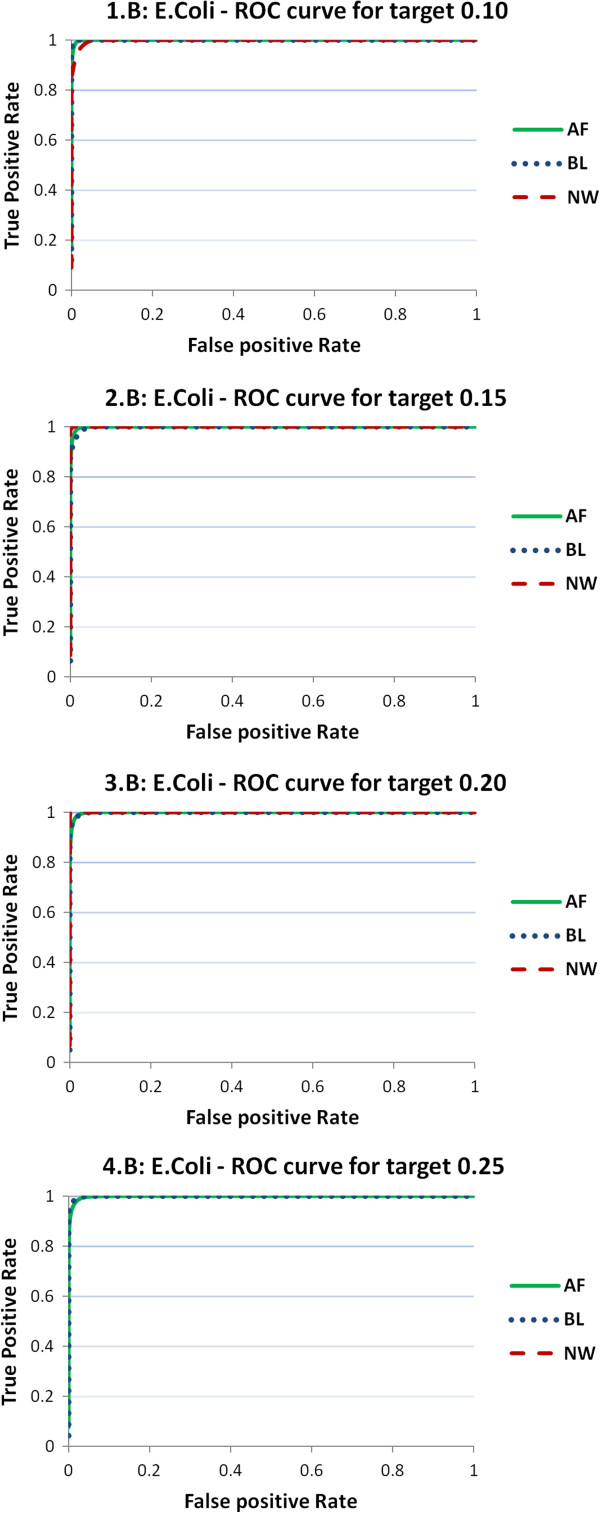
**ROC curves obtained for threshold predictors of E. coli samples.** ROC curves obtained from threshold predictors of four different reference values of the target BT distance (0.10, 0.15, 0.20, 0.25). The charts report the ROC curves for the three predictor distances (NW, BL, AF). Results are provided on samples for E. coli (EA).

**Figure 3:**
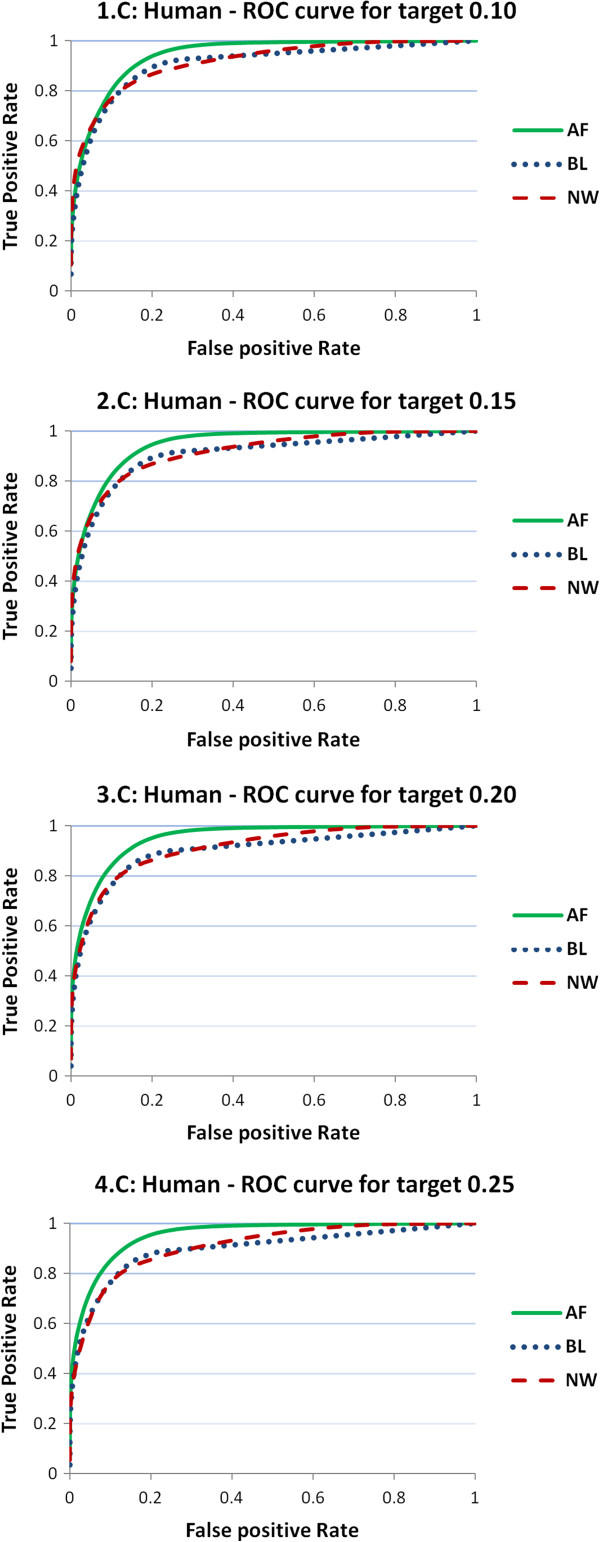
**ROC curves from threshold predictors of human samples.** ROC curves obtained from threshold predictors of four different reference values of the target BT distance (0.10, 0.15, 0.20, 0.25). The charts report the ROC curves for the three predictor distances (NW, BL, AF). Results are provided on samples for human (HA).

**Figure 4:**
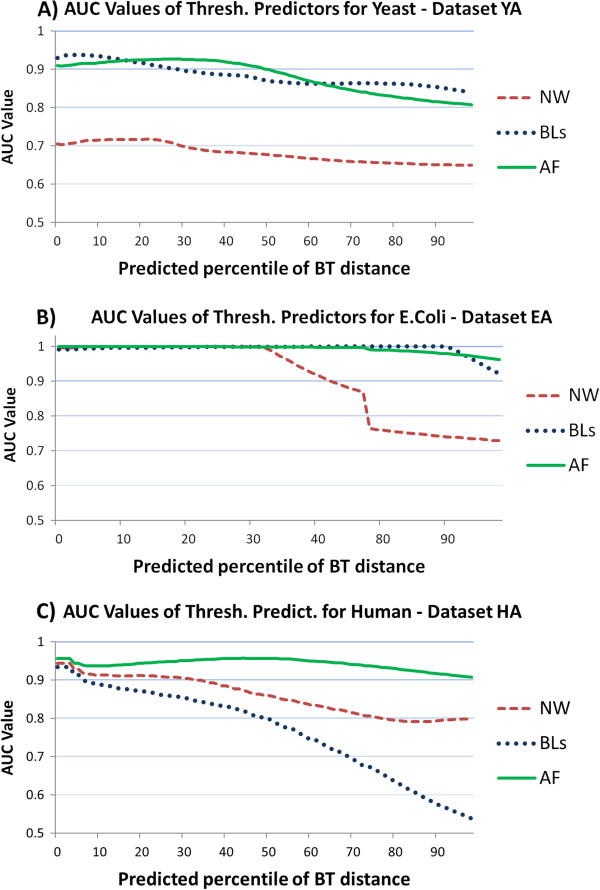
**AUC values for target percentile values.** AUC values for each percentile value of the target BT distance. The three panels report AUC values for the three predictor distances (NW, BL, AF). Results are provided on samples for yeast (panel **A**, YA), E. coli (panel **B**, EA), and human (panel **C**, HA).

We start presenting the ROC curves for the four values 0.10, 0.15, 0.20, and 0.25 of the target distance percentiles that correspond to determined values of BT. In Figures 1, 2 and 3 the ROC curves for predictors NW, BL, and AF are reported for the four reference values and for the three organisms. As one can observe, both AF (green, solid) and BL (blue, dotted) curves perform much better than NW (red, dashed). Figure 1 depicts the ROC curves related to yeast and shows how both AF and BL perform much better than NW with AUC values higher than 0.9, while NW curves have AUC much smaller values (close to 0.7). Figure 2 is related to E. coli and shows a very stable scenario: all the three measures are able to precisely predict BT for all the thresholds reaching values of AUC close to 1. Figure 3 is related to human and shows that AF performs slightly better than NW that in turn performs slightly better than BL. AUC values of AF are close to 0.95, while those of NW are around 0.91, and those of BL range from 0.9 to 0.88. A more comprehensive outlook of the performances of the three predictors can be glanced from the three panels in Figure 4. Here we report the AUC values for all the 100 percentiles of the target distance, for three samples coming from yeast, E. coli, and human. Similar results are obtained when the other five samples from each organism are used (see Additional file 1). The charts clearly show that for all three predictors the precision decreases for higher percentiles (i.e., larger values of the target distance). Lower percentiles (i.e., lower BT) correspond to higher level of overlapping, and we conclude that for these percentiles BT is easily predicted by the three measures.

The higher the percentiles (i.e., the smaller the overlapping), the higher the noise in the prediction will be. AUC values are indeed very high for smaller percentiles with the exception of NW in human. In Figure 4 panel A (yeast), there is evidence that BL and AF have both good performances for all the percentiles in terms of AUC, showing values higher than 0.9, until percentile 30, and anyway values higher than 0.8 for the last percentiles. BL performs slightly better than AF until the percentile 20, then AF is better until the percentile 60 and again BL is better until the last percentiles. NW AUC values range from 0.72 until 0.68 showing again a light decreasing slope. Figure 4 panel B (E. coli) shows - as previously highlighted - that the three measures have very good performances for this organism, with AUC values close to 1 until percentile 33. This means that all the three measures are able to correctly predict BT. From the percentile 33 AUC values of NW significantly decrease, while those of AF and BL are still close to 1 until the percentile 80, when they slowly decrease keeping anyway values higher than 0.9. In Figure 4 panel C (human) it can be observed that the three curves start from an almost common AUC value, around 0.95, but diverge with increasing percentiles. AF has the best performance keeping its AUC values higher than 0.9, then NW decreasing until 0.85, and finally BL falling down to 0.55.

### Cross validation performances of the AFthreshold predictor

The results discussed above show that the BL and AF predictors perform well when they are evaluated on the same sample that has been used to train the predictors reference values. It is more interesting to verify if the relation between the predictor and the target distance, derived from a read pairs sample, maintains its validity also on other samples that were not used to train the method.

**Figure 5:**
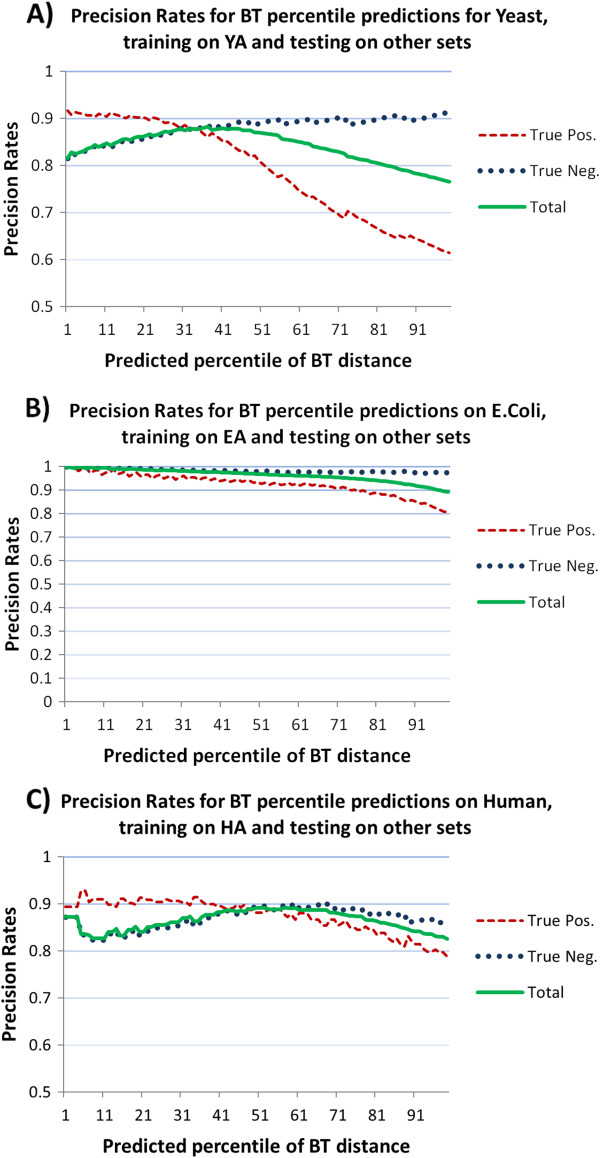
**Cross validation of threshold predictors.** Cross Validation precision rates of threshold predictors for each percentile value of the target BT distance; the panels report precision rates on positive, negative, and total. Results are provided on samples for yeast (panel **A**, YA used for training), E. coli (panel **B**, EA used for training), and human (panel **C**, HA used for training).

We restrict this analysis to the AF predictor and test the threshold predictor rules derived from one sample on the other five samples of the same organism, in a cross validation scheme. The results are summarized in Tables 7, 8 and 9 for yeast, E. coli, and human samples, respectively. The positive and negative precision rates are reported for the four reference values of the target distance already used in Figures 1, 2 and 3 (0.10, 0.15, 0.20, and 0.25), for all the combinations of the cross validation scheme. In Figure 5 we plot the same positive, negative, and total precision rates for all the 100 reference values over a single sample (Panel A for yeast, Panel B for E. coli, and Panel C for human). Similar results have been obtained also for the other five samples of the three organisms.

**Table 7:** **True positive (TP) and true negative (TN) rates for reference values of target distance BT when predicted by AF, for yeast**

Set used for	0.105		0.15		0.205		0.25	
training the predictor	TP [%]	TN [%]	TP [%]	TN [%]	TP [%]	TN [%]	TP [%]	TN [%]
YA	90.26	85.05	90.00	85.80	89.13	86.76	87.96	87.57
YB	90.03	85.23	89.75	86.01	89.69	86.20	87.94	87.61
YC	90.01	85.28	89.72	86.06	88.44	87.34	87.39	88.09
YD	90.23	85.03	89.74	86.03	89.12	86.76	88.58	86.94
YE	90.73	84.52	90.64	85.11	89.05	86.77	87.87	87.57
YF	92.22	70.50	91.41	70.48	91.38	71.96	89.95	73.30
**Average**	90.58	82.60	90.21	83.25	89.47	84.30	88.28	85.18

Predictor is trained on one set and tested over the other 5 sets.

**Table 8:** **True positive (TP) and true negative (TN) rates for reference values of target distance BT when predicted by AF, for E. coli**

Set used for	0.105		0.15		0.205		0.25	
training the predictor	TP [%]	TN [%]	TP [%]	TN [%]	TP [%]	TN [%]	TP [%]	TN [%]
EA	97.00	99.09	96.38	98.78	95.43	98.66	94.61	98.60
EB	99.08	98.14	98.90	97.43	98.54	96.92	97.97	96.78
EC	100.00	96.11	99.98	94.75	99.98	93.48	99.96	92.56
ED	98.67	98.47	98.30	97.93	97.95	97.49	97.49	97.21
EE	98.31	98.69	97.42	98.39	97.15	97.97	96.57	97.84
EF	95.46	99.45	94.15	99.35	93.46	99.19	91.43	99.35
**Average**	98.09	98.32	97.52	97.77	97.09	97.29	96.34	97.06

Predictor is trained on one set and tested over the other 5 sets.

**Table 9:** **True positive (TP) and True negative (TN) rates for reference values of target distance BT when predicted by AF, for human**

Set used for	0.105		0.15		0.205		0.25	
training the predictor	TP [%]	TN [%]	TP [%]	TN [%]	TP [%]	TN [%]	TP [%]	TN [%]
HA	91.05	82.53	91.24	83.24	90.96	84.74	90.19	86.25
HB	94.34	78.82	94.34	79.58	93.67	81.78	94.41	81.35
HC	89.27	84.35	90.15	84.32	88.92	86.24	89.77	86.63
HD	89.32	84.28	90.17	84.27	89.36	86.24	88.99	87.24
HE	89.12	84.22	89.57	84.53	89.02	86.37	87.99	87.82
HF	84.11	85.02	85.47	83.32	84.19	84.04	82.04	85.48
**Average**	89.53	83.20	90.16	83.21	89.36	84.90	88.90	85.80

Predictor is trained on one set and tested over the other 5 sets.

Figure 5 shows that in yeast (panel A) positive precision rate ranges from a percentage of 88.28 (BT = 0.25) until 90.58 (BT = 0.105) while the negative precision rate ranges from 82.60 (BT = 0.105) until 85.18 (BT = 0.25). In E. coli (panel B) both positive and negative precision rates show values always higher than 96.34. In human (panel C) positive precision rate always is around a percentage of 90 and negative precision rate ranges from 83.07 (BT = 0.105) and 85.98 (BT = 0.25).

Tables 7, 8 and 9 show positive, negative, and total precision rates for all the percentiles in the three organisms, revealing, as expected, that for E. coli (Table 8) AF has very good performances also in the cross validation (total precision rate is always higher than 0.9), with a slight decreasing slope for the higher percentiles. In human and yeast (Table 9 and Table 7, respectively), we have globally good performances, with a total precision rate ranging from 0.8 to 0.9.

The results described in Tables 7, 8 and 9 and Figure 5 confirm indeed that the AF predictor performs very well also in the cross validation scheme and exhibits good generalization capabilities. The parallel analysis conducted on the other two candidate predictors shows similar performances of BL and much poorer performances of NW (results shown in Additional file 1).

### Final discussion

The results clearly show the efficacy of the alignment-free distance in estimating a good read-to-read distance measure. The performances of AF in predicting BT are better than NW and at least comparable to BL, but the advantage of using AF is clear: it is linear in the size of the input and has a lower computational time. Indeed, as already discussed in subsection Computational time analysis of the threshold predictors, AF is much faster than NW and BL. As reported above, the prediction power of the three measures depends on the organism we consider, and we believe that this issue deserves further analysis and discussion.

We analyze two eukariotic genomes (yeast and human) and one bacterial one (E. coli); there is evidence that the performances of all the three predictors are globally much better in E. coli. This may be due to the nature of bacterial genomes, which are mostly composed of coding sequences, making easier to recognize overlapping regions and reducing the noise due to low complexity regions present in the intergenic eukariotic portions of genome.

An additional fact that deserves attention is that the distance-based on global alignment (NW), generally performs poorly with respect to the one based on local alignment (BL); the alignment-free distance (AF) seems to compare well with the local alignment one, despite it is based on the evaluation of the whole sequence, overcoming the bias that may derive from requiring the global alignment of the two reads.

Such a consideration is somehow strengthened by the different performances obtained on reads of different sizes; we recall that reads from human are smaller (average size 75 bases) than yeast and E. coli (100 and 235 bases, respectively); such a difference may explain the improved performances of the NW distance in human, as with shorter reads the advantage of local versus global alignment is reduced.

## Conclusions

In this paper, we described and tested a method to compare NGS DNA reads based on an alignment-free distance. We compared our method with respect to Blast and Needleman-Wunsch algorithms, which rely on an alignment-based approach. We designed our experiments in order to measure the potential contribution of the method in filtering DNA reads and speed up an assembly process.

We showed that the alignment-free distance outperformed the two aligned-based ones both in terms of computational time and of prediction performance, and conclude that an alignment-free distance may be used effectively for read pairs comparison.

In future, we plan to extend the reads comparison with other competitive methods and also with other alignment-free distances. The results shown in this paper are considered as a starting point to derive more efficient sequence similarity assessments methods for DNA reads obtained from NGS sequencing.

Finally, read pairs comparison based on alignment-free distances may be conveniently used in future for DNA assembly [34] given its considerable speed, as well as for reads classification [35], e.g., in metagenomics.

## Electronic supplementary material

Additional file 1: **Microsoft Excel 2010 spreadsheet that contains additional charts and data that are not shown in the paper.** It is divided into 6 sheets: • Sheet SP1 AUC for yeast samples contains the AUC plots for the different percentiles of the target distance and the different predictors for the six different samples of read pairs from yeast (YA-YF). • Sheet SP2 AUC for E. coli samples contains the AUC plots for the different percentiles of the target distance and the different predictors for the six different samples of read pairs from E. coli (EA-EF). • Sheet SP3 AUC for human samples contains the AUC plots for the different percentiles of the target distance and the different predictors for the six different samples of read pairs from human (HA-HF). • Sheet SP4 NW-BL-AF test precision contains the precision of the three predictors for the different percentiles of the target distance, expressed in terms of true positive, true negative and total precision rates, for the HA read pairs sample of human. • Sheet SP5 test-human contains the detailed test results of the cross validation on the six samples of human. • Sheet SP6 test-ecoli contains the detailed test results of the cross validation on the six samples of E. coli. (XLSX 1012 KB)

## References

[1] Eisenstein M (2012). The battle for sequencing supremacy. Nat Biotechnol.

[2] Liu L, Li Y, Li S, Hu N, He Y, Pong R, Lin D, Lu L, Law M (2012). Comparison of next-generation sequencing systems. J Biomed Biotechnol.

[3] Metzker ML (2010). Sequencing technologies - the next generation. Nat Rev Genet.

[4] Earl D, Bradnam K, John JS, Darling A, Lin D, Fass J, Yu HOK, Buffalo V, Zerbino DR, Diekhans M, Ariyaratne PN, Sung W-K, Ning Z, Haimel M, Simpson JT, Fonseca NA, Birol I, Docking TR, Ho IY, Rokhsar DS, Chikhi R, Lavenier D, Chapuis G, Naquin D, Maillet N, Schatz MC, Kelley DR, Phillippy AM, Koren S, Nguyen N (2011). Assemblathon 1: a competitive assessment of de novo short read assembly methods. Genome Res.

[5] Bradnam KR, Fass JN, Alexandrov A, Baranay P, Bechner M, Birol I, Boisvert S, Chapman JA, Chapuis G, Chikhi R, Chitsaz H, Chou W-C, Corbeil J, Fabbro CD, Docking TR, Durbin R, Earl D, Emrich S, Fedotov P, Fonseca NA, Ganapathy G, Gibbs RA, Gnerre S, Godzaridis E, Goldstein S, Haimel M, Hall G, Haussler D, Hiatt JB, Ho IY (2013). Assemblathon 2: evaluating de novo methods of genome assembly in three vertebrate species. GigaScience.

[6] Nagarajan N, Pop M (2009). Parametric complexity of sequence assembly: theory and applications to next generation sequencing. J Comput Biol.

[7] Blazewicz J, Bryja M, Figlerowicz M, Gawron P, Kasprzak M, Kirton E, Platt D, Przybytek J, Swiercz A, Szajkowski L (2009). Whole genome assembly from 454 sequencing output via modified dna graph concept. Comput Biol Chem.

[8] Compeau PEC, Pevzner PA, Tesler G (2011). How to apply de bruijn graphs to genome assembly. Nat Biotechnol.

[9] Birol I, Jackman SD, Nielsen CB, Qian JQ, Varhol R, Stazyk G, Morin RD, Zhao Y, Hirst M, Schein JE, Horsman DE, Connors JM, Gascoyne RD, Marra MA, Jones SJ (2009). De novo transcriptome assembly with abyss. Bioinformatics.

[10] Zerbino DR, Birney E (2008). Velvet: algorithms for de novo short read assembly using de bruijn graphs. Genome Res.

[11] Luo R, Liu B, Xie Y, Li Z, Huang W, Yuan J, He G, Chen Y, Pan Q, Liu Y, Tang J, Wu G, Zhang H, Shi Y, Liu Y, Yu C, Wang B, Lu Y, Han C, Cheung DW, Yiu S-M, Peng S, Xiaoqian Z, Liu G, Liao X, Li Y, Yang H, Wang J, Lam T-W, Wang J (2012). Soapdenovo2: an empirically improved memory-efficient short-read de novo assembler. Gigascience.

[12] Miller JR, Koren S, Sutton G (2010). Assembly algorithms for next-generation sequencing data. Genomics.

[13] Vinga S, Almeida J (2003). Alignment-free sequence comparison-a review. Bioinformatics.

[14] Polychronopoulos D, Weitschek E, Dimitrieva S, Bucher P, Felici G, Almirantis Y (2014). Classification of selectively constrained dna elements using feature vectors and rule-based classifiers. Genomics.

[15] Li M, Vitnyi PMB (2008). An Introduction to Kolmogorov Complexity and Its Applications.

[16] Almeida JS, Vinga S (2002). Universal sequence map (usm) of arbitrary discrete sequences. BMC Bioinf.

[17] Giancarlo R, Scaturro D, Utro F (2009). Textual data compression in computational biology: a synopsis. Bioinformatics.

[18] Kuksa P, Pavlovic V (2009). Efficient alignment-free dna barcode analytics. BMC Bioinf.

[19] Hide W, Burke J, Da Vison DB (1994). Biological evaluation of d2, an algorithm for high-performance sequence comparison. J Comput Biol.

[20] Teeling H, Meyerdiekers A, Bauer M, Glöckner FO (2004). Application of tetranucleotide frequencies for the assignment of genomic fragments. Environ Microbiol.

[21] Pride DT, Meinersmann RJ, Wassenaar TM, Blaser MJ (2003). Evolutionary implications of microbial genome tetranucleotide frequency biases. Genome Res.

[22] Teeling H, Waldmann J, Lombardot T, Bauer M, Glöckner FO (2004). Tetra: a web-service and a stand-alone program for the analysis and comparison of tetranucleotide usage patterns in dna sequences. BMC Bioinf.

[23] Langmead B, Trapnell C, Pop M, Salzberg SL (2009). Ultrafast and memory-efficient alignment of short dna sequences to the human genome. Genome Biol.

[24] Langmead B, Salzberg SL (2012). Fast gapped-read alignment with bowtie 2. Nat Methods.

[25] Needleman SB, Wunsch CD (1970). A general method applicable to the search for similarities in the amino acid sequence of two proteins. J Mol Biol.

[26] **NeoBio: Bioinformatics Algorithms in Java** [ http://neobio.sourceforge.net/]

[27] Altschul S, Gish W, Miller W, Myers E, Lipman D (1990). Basic local alignment search tool. J Mol Biol.

[28] **Blast Package Version 2.2.25–7** http://packages.ubuntu.com/precise/ncbi-blast+

[29] Fawcett T (2006). An introduction to roc analysis. Pattern Recognit Lett.

[30] **NCBI Sequence Read Archive** http://www.ncbi.nlm.nih.gov/sra

[31] **E. Coli Reads Source** http://petang.cgu.edu.tw/Bioinfomatics/Lecture/0_HTS/08/HTS_E08.pdf

[32] **Yeast Bowtie Index** http://ftp.ccb.jhu.edu/pub/data/bowtie_indexes/s_cerevisiae.ebwt.zip

[33] **E. Coli Bowtie Index** http://ftp.ccb.jhu.edu/pub/data/bowtie_indexes/e_coli.ebwt.zip

[34] **Dazzler Assembler for PacBio Reads** http://www.homolog.us/blogs/blog/2014/02/14/dazzle-assembler-pacbio-reads-gene-myers/

[35] Song K, Ren J, Zhai Z, Liu X, Deng M, Sun F (2013). Alignment-free sequence comparison based on next generation sequencing reads. J Comput Biol.

